# Human Platelets Internalize Pregnancy-Specific Glycoprotein-1 (PSG1)

**DOI:** 10.1055/a-2618-4817

**Published:** 2025-06-10

**Authors:** Ejaife O. Agbani, Lorraine Chow, Joshua Nicholas, Ibukun Akinrinade, Nancy Soliman, Donna M. Slater, Pavel Davizon-Castillo, Gabriela Dveksler

**Affiliations:** 1 Department of Physiology and Pharmacology, Cumming School of Medicine, University of Calgary, Alberta, Canada; 2 McCaig Institute for Bone and Joint Health, University of Calgary, Alberta, Canada; 3 Department of Anaesthesiology, Perioperative and Pain Medicine, Cumming School of Medicine, University of Calgary, Alberta, Canada; 4 Department of Obstetrics and Gynecology, Cumming School of Medicine, University of Calgary, Alberta, Canada; 5 Bloodworks Research Institute, Seattle, Washington, United States; 6 Department of Pathology, School of Medicine, Uniformed Services University of the Health Sciences, Bethesda, Maryland, United States

**Keywords:** imaging, platelet physiology, pregnancy, PSG1, pregnancy-specific glycoproteins

## Abstract

It has been long suggested that the placenta “educates” maternal platelets to contribute to a healthy pregnancy. Several studies have also demonstrated unique changes in platelet function and ultrastructure during pregnancy, some of which may drive hypertensive complications of pregnancy. One of the few proteins that are differentially found in the plasma of pregnant females when compared with non-pregnant females and males are the members of the pregnancy-specific glycoprotein (PSG) family, and PSG1 is one of the highest expressed and best characterized of all human PSGs. Because PSGs are secreted into the maternal circulation (by the trophoblast cells of the placenta), platelets may be picking up placental exosomes containing PSGs. Also, platelets may directly incorporate circulating PSGs, which are found in high concentration, as has been shown for other serum proteins, including fibrinogen. In this image report, we have utilized a state-of-the-art high-resolution imaging approach to examine the interactions of labeled recombinant PSG1 with non-permeabilized human platelets. Strikingly, we observed that human platelets internalize PSG1 and express PSGs during pregnancy.

Pregnancy is associated with changes in platelet ultrastructure and function, 1 and it has been suggested that the placenta “educates” maternal platelets to contribute to a healthy pregnancy. 2 The members of the pregnancy-specific glycoprotein (PSG) family are secreted from the syncytiotrophoblast layer of the placenta to the maternal circulation and are one of the few proteins found in the plasma of pregnant females that are absent in non-pregnant females and males. 3 There are 10 protein-coding human PSG genes (PSG1–PSG9, PSG11) 4 and all have been shown to activate latent TGF-β. 5 Previous studies by our group and others have detected PSGs in platelet lysates 3 and releasates, 6 which was unexpected considering their placental origin. Furthermore, PSG1 has been reported to bind platelet integrin α _IIb_ β _3_ and inhibit fibrinogen binding. 7 Here, we provide visual evidence which suggests platelets can interact, bind, and internalize plasma PSGs using recombinant PSG1, a highly expressed and well characterized of all human PSGs. 8 PSG1 was generated from the supernatant of stably transfected CHO-K1 single-cell clone established in our laboratory and grown in hollow fiber cartridge bioreactors (Fiber Cell Systems, Frederick, MD, USA), as previously described. 9 10 To fluorescently label PSG1, we used the Alexa Fluor 647 labeling kit (Molecular Probes, Thermo Fisher Scientific), following the manufacturer's instructions. We utilized a high-resolution fluorescence imaging approach to examine the interactions and sub-cellular localization of PSG1-labeled Alexa-Fluor 647 with non-permeabilized platelets derived from non-pregnant females and participants with a healthy pregnancy. Alexa-Fluor 405-conjugated CD42b/GP1b α antibody (Bio-Techne Canada, GP1bα, **Blue** ) was used as a platelet marker in combination with size discrimination. In Fig. 1 , the images in Panel A are selected z-plane images from z-stack images of platelets separated by 0.25 µm. Panel B of Fig. 1 shows platelets in panel A in an XYZ orientation, and the insert shows extended focus (2D projection) images of the same platelets. In Panel C ( Fig. 1 ), we show in **Ci** , images of the spatiotemporal interaction of labeled PSG1 with GPIbα marked platelets. The time points for **Ci** (f1–f70) correspond to 15 to 100 seconds. **Cii** shows quantification of Manders' colocalization coefficient of PSG1 with GPIbα labeled platelets, and the kinetics of PSG1 interaction with GPIbα labeled platelets adhering to serum albumin ( **Ciii** ) and fibrinogen 200 µg/mL coating ( **Civ** ). The data in **Cii** and **Ciii** are from eight donors. Manders' coefficients, M1 and M2, indicate the extent of PSG1 and GP1b α colocalization. M1 represents the proportion of the PSG1 signal that colocalizes with the platelet marker GP1b α signal, and M2 indicates the colocalization of GP1b α with PSG1. In parallel experiments using an anti-PSG antibody (BAP3; sc-59348, Santa Cruz), we observed PSG on the membrane and inside the platelets of all pregnant women examined ( *n* = 8; not shown). Platelets were derived from pregnant women during the 3rd trimester (gestational age 224–272 days), and 2 to 14 days before delivery. Furthermore, labeled recombinant PSG1 ( **Green** ) was internalized by platelets from non-pregnant females ( **Panels A–C** ) and localized within the cytosol and on the plasma membrane ( **Panels A–C** ). PSG1 internalization occurred in platelets in quiescent states adhering to serum albumin ( **Ciii** ), and the uptake was enhanced in the presence of fibrinogen ( **Civ** ). The associated supplementary data ( Videos 1 and 2 , available in the online version only) show the spatial distribution of labeled PSG1 as the platelet XY planes images are examined over Z heights ( Video 1 , available in the online version only). Video 2 (available in the online version only) captures the interaction of labeled PSG1 with platelets over time. Images were captured at Nyquist using a Nikon A1R laser scanning confocal microscope via Nikon NIS-Elements imaging software and an oil immersion Plan Apo Lambda objective lens (60x; Numerical Aperture: 1.4; Working Distance: 0.13 mm). Image resolution was improved by the restoration complement of the Volocity® imaging Software and analyzed using the same software (Quorum Technologies Inc. Canada). Scale bars: 3 μm ( **A, B** ).

**Fig. 1:**
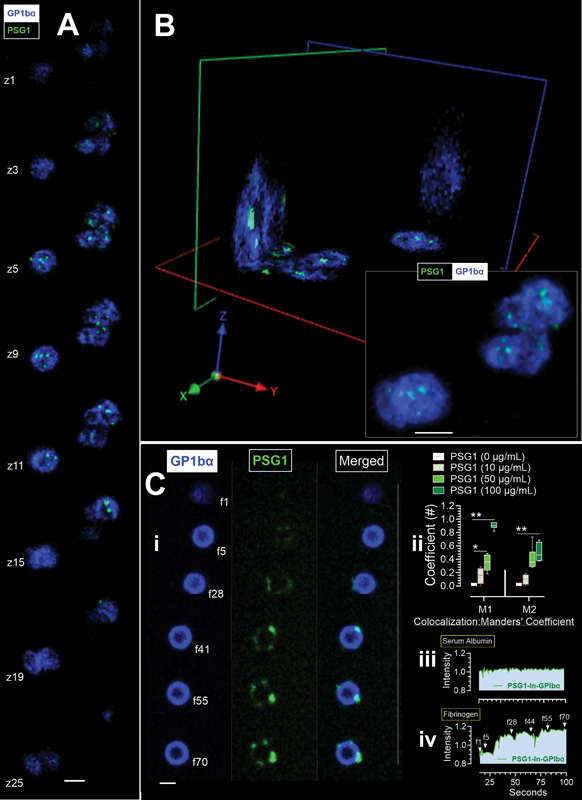
Human Platelets Internalise Pregnancy-Specific Glycoprotein-1 (PSG1).

**Video 1** Spatial localisation of PSG1 in non-permeabilised human platelets.


**Video 2** Kinetics of PSG1 interaction with non-permeabilised human platelets.


## References

[1] Agbani E O Chow L Nicholas J Overexpression of facilitative glucose transporter-3 and membrane procoagulation in maternal platelets of preeclamptic pregnancy J Thromb Haemost 2023 21 07 1903 1919 36963633 10.1016/j.jtha.2023.03.014

[2] Guettler J Forstner D Gauster M Maternal platelets at the first trimester maternal-placental interface—small players with great impact on placenta development Placenta 2022 125 61 67 34920861 10.1016/j.placenta.2021.12.009

[3] de Almeida L GN Young D Chow L Proteomics and metabolomics profiling of platelets and plasma mediators of thrombo-inflammation in gestational hypertension and preeclampsia Cells 2022 11 08 1256 35455936 10.3390/cells11081256 PMC9027992

[4] Moore T Dveksler G S Pregnancy-specific glycoproteins: complex gene families regulating maternal-fetal interactions Int J Dev Biol 2014 58(2-4):273 280 25023693 10.1387/ijdb.130329gd

[5] Warren J Im M Ballesteros A Activation of latent transforming growth factor-β1, a conserved function for pregnancy-specific beta 1-glycoproteins Mol Hum Reprod 2018 24 12 602 612 30371828 10.1093/molehr/gay044 PMC6262632

[6] Szklanna P B Parsons M E Wynne K The platelet releasate is altered in human pregnancy Proteomics Clin Appl 2019 13 03 e1800162 30318839 10.1002/prca.201800162

[7] Shanley D K Kiely P A Golla K Pregnancy-specific glycoproteins bind integrin αIIbβ3 and inhibit the platelet-fibrinogen interaction PLoS One 2013 8 02 e57491 23469002 10.1371/journal.pone.0057491 PMC3585349

[8] Moore T Williams J M Becerra-Rodriguez M A Dunne M Kammerer R Dveksler G Pregnancy-specific glycoproteins: evolution, expression, functions and disease associations Reproduction 2022 163 02 R11 R23 35007205 10.1530/REP-21-0390

[9] Ballesteros A Mentink-Kane M M Warren J Kaplan G G Dveksler G S Induction and activation of latent transforming growth factor-β1 are carried out by two distinct domains of pregnancy-specific glycoprotein 1 (PSG1) J Biol Chem 2015 290 07 4422 4431 25548275 10.1074/jbc.M114.597518 PMC4326847

[10] Rattila S Dunk C EE Im M Interaction of pregnancy-specific glycoprotein 1 with integrin Α5β1 is a modulator of extravillous trophoblast functions Cells 2019 8 11 1369 31683744 10.3390/cells8111369 PMC6912793

